# The genetics‐morphology‐behavior trifecta: Unraveling the single greatest limitation affecting our understanding of chondrichthyan evolution

**DOI:** 10.1002/ece3.10204

**Published:** 2023-06-17

**Authors:** Joel H. Gayford

**Affiliations:** ^1^ Department of Life Sciences Imperial College London London UK; ^2^ Shark Measurements London UK

**Keywords:** batoidea, ecomorphology, Elasmobranchii, holocephali, natural selection, quantitative genetics, shark

## Abstract

Sharks, rays, and chimaera form the clade Chondrichthyes, an ancient group of morphologically and ecologically diverse vertebrates that has played an important role in our understanding of gnathostome evolution. Increasingly, studies seek to investigate evolutionary processes operating within the chondrichthyan crown group, with the broad aim of understanding the driving forces behind the vast phenotypic diversity observed among its constituent taxa. Genetic, morphological, and behavioral studies have all contributed to our understanding of phenotypic evolution yet are typically considered in isolation in the context of Chondrichthyes. In this viewpoint, I discuss why such isolation is prevalent in the literature, how it constrains our understanding of evolution, and how it might be overcome. I argue that integrating these core fields of organismal biology is vital if we are to understand the evolutionary processes operating in contemporary chondrichthyan taxa and how such processes have contributed to past phenotypic evolution. Despite this, the necessary tools to overcome this major limitation already exist and have been applied to other taxa.

## Graphical abstract

Major knowledge gaps exist regarding Chondrichthyan trait evolution. Lack of integration between key fields of organismal biology is the main driver of this uncertainty. Increase in research effort and application of methodologies used frequently in other clades may be sufficient to overcome this lack of integration.

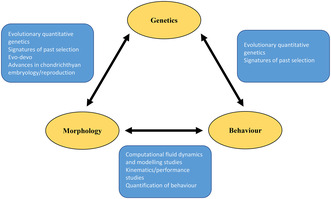

## 1 OVERVIEW

Sharks, rays, and chimaera form the clade Chondrichthyes, a group of cartilaginous fishes thought to have first arisen during the Paleozoic and thus to have survived all five mass extinctions (Guinot & Cavin, 2016; Soldo, 2013; Stiassny et al., 2004). Over the course of its long history the clade has undergone major transitions in diversity (Grogan et al., 2012; Guinot & Cavin, 2016; Kriwet & Benton, 2004; Kriwet & Klug, 2008) but remains a morphologically and ecologically diverse component of modern ecosystems (Cailliet et al., 2005; Compagno, 1990; Kolmann et al., 2022; Stein et al., 2018), performing various important ecological functions (Flowers et al., 2021; Heupel et al., 2014; Navia et al., 2010). Chondrichthyan taxa have long been influential in studies of vertebrate, and particularly gnathostome evolution (Gillis & Shubin, 2009; Smith, 2003). Due to their phylogenetic position, chondrichthyans have previously been utilized to represent the ancestral gnathostome condition when considering evolutionary transitions in specific morphological/developmental characters (Gillis et al., 2013; Mallatt, 1996), although given that many character states in extant chondrichthyans are likely derived, the value of such an approach is debatable. Chondrichthyan taxa have also been used as case studies for understanding the evolution of phenomena such as genetic conflict and sexual dimorphism (Crespi & Semeniuk, 2004; DiBattista et al., 2008; Gayford, 2023). Unfortunately, chondrichthyans are currently facing a global extinction crisis (Stein et al., 2018), with over 300 species vulnerable to extinction and many more severely under‐studied (Dulvy et al., 2021). For this reason, it has never been more important to improve our understanding of chondrichthyan evolution—particularly in the context of contemporary populations.

The fields of genetics, morphology, and behavior have each played important roles in our understanding of evolution. Phylogenetics has revolutionized our understanding of phenotypic evolution and interrelationships (Didier, 1992; Last et al., 2016; Lee & Palci, 2015; Naylor et al., 2005), whereas morphological and behavioral studies have both provided insight into how organisms interact with other components of the ecosystem (Wainwright, 1994, 1996) and the evolutionary processes operating within natural populations (Davies et al., 2012; Le Roy et al., 2019; Owens, 2006). While important in isolation, it is the interplay between genetics, morphology, and behavior that is most significant for understanding evolutionary processes, their phenotypic consequences, and how such phenotypes engage with the wider ecological community (Arnold, 1983; Lewontin & Krimpas, 2000; Owens, 2006; Wainwright, 1994). Regrettably, studies of chondrichthyan evolution rarely unify these concepts, fundamentally limiting our ability to identify and understand the nature of evolutionary processes operating in this clade.

Here, I explain how this failure to integrate between core fields of organismal biology has significantly hampered our understanding of chondrichthyan evolution, both past and present. I provide reasons for such failures as well as potential solutions and examples of their application in other taxa. I suggest that integration of genetics, morphology, and behavioral studies is crucial to our understanding of phenotypic evolution in both past and present chondrichthyan populations.

## 2 LINKING MORPHOLOGY TO GENETICS

Comparative studies of vertebrate anatomy and morphogenesis have contributed significantly to our understanding of evolutionary history and taxonomic interrelationships (Lee & Palci, 2015), and this is no different in Chondrichthyes (Naylor et al., 2005; Shirai, 1996). Despite this, the interrelationships of various chondrichthyan subclades have long been debated (Klug, 2010; Naylor et al., 2005; Qiao et al., 2016) and it is only relatively recently, upon the development of integrated morphological and molecular phylogenies with high taxonomic coverage (Naylor et al., 2005, 2012; Stein et al., 2018), that these controversies have been resolved. Morphological studies remain central to our understanding of both chondrichthyan interrelationships (Da Silva et al., 2018, 2023; Soares & Mathubara, 2022; Villalobos‐Segura et al., 2022) and character evolution (Da Silva & De Carvalho, 2015; Jambura et al., 2023), with the major benefit of allowing the incorporation of fossil taxa—the only direct evidence of past evolutionary change—in analyses. It is abundantly clear however that incorporating both sources of data into phylogenetic and evolutionary hypotheses greatly improves our understanding of evolution, morphological, or otherwise. Phylogenetics is likely the most universally applicable integration of genetics and morphology, however increasingly evolutionary‐developmental (evo‐devo) studies and evolutionary genetics are being used to uncover the genetic basis of morphological traits (Abzhanov et al., 2002; Mallarino & Abzhanov, 2012) and the selective regimes under which they have evolved (Ho et al., 2017; Rolland et al., 2018). Evo‐devo studies targeting chondrichthyan taxa are present in the literature and have revolutionized our understanding of the acquisition of certain morphological characters (Marlétaz et al., 2023), yet are restricted to a small number of morphological characters in a minute proportion of extant species (Gillis et al., 2013; Gillis & Shubin, 2009; Marlétaz et al., 2023). Besides this, we have extremely minimal knowledge of the genetic and developmental underpinnings of morphological variation in Chondrichthyes.

This of course represents a knowledge gap in itself, but also fundamentally constrains our ability to understand morphological evolution within Chondrichthyes. One of the main benefits of past morphological studies is that they allow not only for the use of comparative phylogenetics methods (e.g., Pimiento et al., 2019), but can incorporate fossil taxa, enabling the sequential and stepwise nature of morphological character transitions over macroevolutionary time to be unraveled (Da Silva & De Carvalho, 2015; Jambura et al., 2023). Critically, in the absence of either detailed knowledge regarding the genetic/developmental basis of morphological structures or the use of an ecomorphological approach (as described below), it is extremely difficult to infer anything about the nature of the evolutionary processes (including the dominant mode of selection) driving these character transitions. Thus whilst the mechanistic process of morphological evolution can be described in detail, the selective regimes favoring specific evolutionary trajectories remain poorly understood. Of particular relevance to morphological evolution, lack of integration between genetic and morphological studies constrains understanding of the extent to which evolutionary modularity/integration influences chondrichthyan morphological evolution. Both are known to have played important roles in other clades, and recently studies have begun to assess the potential for modular/integrated morphological evolution in some chondrichthyan taxa (Klingenberg, 2008; López‐Romero et al., 2020). It is now understood that the extent of evolutionary integration in morphological structures depends upon several factors including the extent of shared gene regulatory networks, genetic linkage at loci underlying morphological variation, and ecological trait correlations (Brakefield, 2006; Lande & Arnold, 1983; Saltz et al., 2017). It is thus effectively impossible to understand the factors underlying modular/integrated morphological evolution without the incorporation of evo‐devo and/or quantitative genetic studies, which is critical given that each of these factors has distinct evolutionary implications. Understanding the structural level at which selection is acting upon morphology is of unparalleled importance, and for this reason alone a significant increase in evo‐devo and quantitative genetic studies is warranted.

Some studies utilize an ecomorphological approach to chondrichthyan morphological evolution in an attempt to uncover the adaptive value of morphology (Gayford et al., 2023), under the assumption that ecological selection is dominant and has shaped the evolution of morphological structures (Andrew Barr, 2018). In this case, the lack of integration between morphological and genetic studies is equally (if not more) problematic, as these studies typically negate entirely the potential role of constraint in morphological evolution (Gayford et al., 2023). The importance of these constraints to morphological evolution in other taxa is well known (Beldade et al., 2002; Wagner, 1996), however, such an understanding relies upon knowledge of the genetic architectures or gene regulatory networks underlying morphology (Davidson & Erwin, 2006; Hegmann & Possidente, 1981). Evolutionary constraints such as genetic correlations can substantially alter the pace of evolution by modulating the response to selection (Crespi, 2000; Greenbury et al., 2016), whilst others such as lack of additive variance can make ‘optimal’ genotypes effectively unattainable (Hansen et al., 2003) or result in maladaptive evolution (Crespi, 2000). There is debate surrounding the extent to which short‐term genetic constraints influence long‐term evolution (Dooren, 2020; Hadfield et al., 2007), however, even if we ignore mounting evidence regarding the importance of such constraints to past evolution (Futuyma, 2010), the relevance of these concepts to the immediate future of chondrichthyans and their contemporary evolution is unquestionable: in light of their dire conservation status (Dulvy et al., 2021; Stein et al., 2018), low fecundity, and long generation times (Cailliet et al., 2005), an understanding of the extent to which constraint may influence future morphological adaptation to environmental change should be one of the key priorities of contemporary chondrichthyan evolutionary research.

**FIGURE 1:**
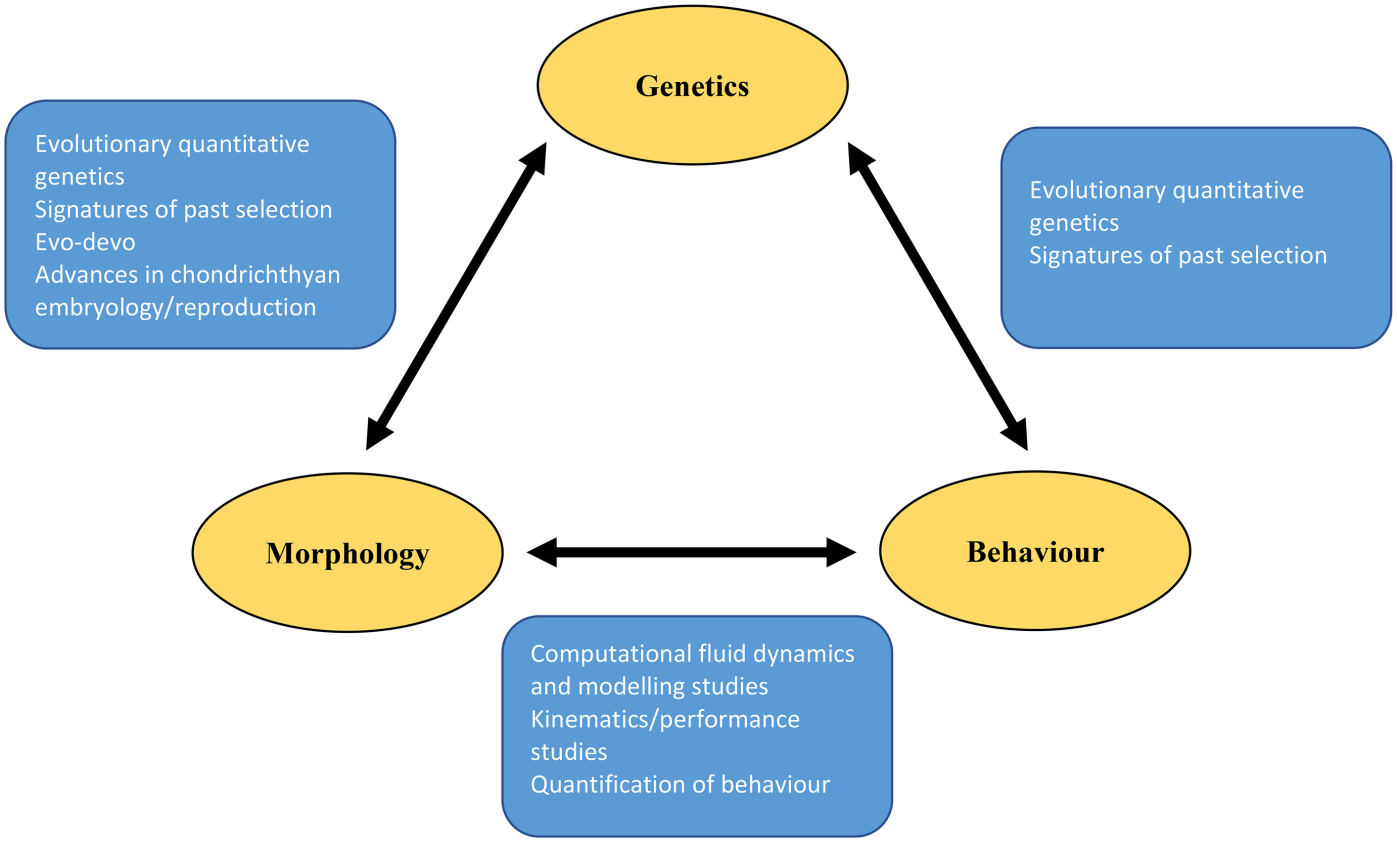
Methodologies that could be utilized to increase integration between genetic, behavioral, and morphological studies of chondrichthyan evolution. This is not an exhaustive list, but rather a selection of approaches that could be implemented in Chondrichthyes without major technological innovation.

There are several explanations for the lack of previous integration between morphological and genetic studies of chondrichthyan evolution. In the case of evo‐devo studies, research effort is among the primary limitations, with only a relatively low number of morphological structures considered from a handful of species (Gillis & Shubin, 2009). Conducting such studies in a greater range of taxa would increase our understanding of the genetic basis of morphological variation within Chondrichthyes and the extent to which trait correlations and evolutionary constraints relating to gene regulatory networks appear to be present (Figure 1). Another major issue prohibiting more widespread usage of evo‐devo studies is the variation in reproductive mode exhibited within Chondrichthyes. Existing studies are restricted to oviparous taxa as they are more amenable to experimental embryonic manipulation and develop freely without any physical connection to maternal tissues. A large number of chondrichthyan taxa utilize placental or aplacental matrotrophy (Awruch, 2015) which is problematic due to the reliance of embryos on maternal provisioning. Moreover, many chondrichthyans have relatively low fecundity (Cortés, 2000), meaning large laboratory populations would need to be maintained in many cases. These issues are unlikely to be insurmountable as evo‐devo studies have been performed on other matrotrophic organisms (Howenstine et al., 2021; Renvoisé & Michon, 2014), however further advances in our understanding of chondrichthyan embryology and reproduction will be required if we wish to extend the taxonomic coverage of evo‐devo studies (Figure 1). Potential advances may relate to cell and tissue culture, external imaging and in‐vivo studies, and artificial manipulation of fecundity in laboratory‐held populations (Braasch et al., 2015; Campos‐Mendoza et al., 2004; Wolf & Ahne, 1982).

Where the genetic basis of traits is known, quantitative genetics can be used to study the evolutionary processes underlying evolution and how they have contributed to observed phenotypic variation (Barton & Turelli, 1989; Roff, 2012). These processes include but are not limited to natural selection, migration, mutation, and genetic drift. Genome mapping and association studies can help unravel potential interactions between each of these factors and elucidate the genetic architecture underlying morphological traits, which in turn provides valuable insight into the role of constraint and genetic correlations (Lee et al., 2016; Mackay, 2004). Quantitative genetic studies of chondrichthyan populations have previously been limited by the costs of genome sequencing, however technological advances are rapidly increasing the accessibility of sequencing technologies (Mardis, 2017). The requirement of pedigree information for quantitative trait loci (QTL) and genome‐wide association studies (GWAS) also provides a limitation to their usage in wild populations (Day‐Williams et al., 2011). Recent advances have softened this requirement, with several routes available for quantitative genetic analyses in wild populations where pedigree information is absent (Johnston et al., 2022). Finally, where the genetic basis of morphological traits is known, signatures of past selection can be detected through phylogenetic analysis (Vitti et al., 2013). This approach utilizes sequence information (typically the rate of non‐synonymous mutations or levels of polymorphism relative to divergence) to uncover molecular signatures consistent with specific modes of selection (Egea et al., 2008; Hudson et al., 1987; Vitti et al., 2013). Importantly, these methodologies are not mutually exclusive, and should all contribute to future research, through which we can improve our understanding of past morphological evolution and future ‘evolvability’ of morphological traits in chondrichthyan taxa.

## 3 LINKING BEHAVIOR TO GENETICS

Whilst there is no consensus definition of behavior in the literature (Levitis et al., 2009), behavioral traits do not differ fundamentally from any other class of trait, and many have been shown to have some degree of genetic underpinning (Bleakley et al., 2010; Fitzpatrick et al., 2005). Studying behavior in extinct species is challenging (Hsieh & Plotnick, 2020), and thus understanding the relevance of behavioral studies to past evolutionary events can be difficult. Nevertheless, integrative studies combining genetics and behavioral ecology are essential to our understanding of the adaptive value (or lack thereof) of behaviors (Penke et al., 2007). This is of paramount importance to our understanding of evolution both past and present given that behavior is the suite of traits by which ecological interactions (both inter and intraspecific) are directly mediated. Unfortunately, our understanding of chondrichthyan behavior (Bres, 1993; Guttridge et al., 2009), let alone the genetic basis of this behavior is severely limited. This hampers our understanding of chondrichthyan evolution in much the same way as a lack of integration between morphology and genetics: without an understanding of the genetic architectures and adaptive landscapes underlying behavioral traits we are fundamentally constrained in our ability to understand behavioral evolution, how it has contributed to the evolution of phenotypic diversity observed in extant taxa, and how it may influence organismal evolution in the face of rapid environmental change. Of particular importance to contemporary populations, it is not possible to evaluate the posited ‘special’ role of behavior in evolution without an understanding of the genetic basis of behavioral traits (Levis & Pfennig, 2016; McGlothlin & Brodie III, 2009).

The importance of behavior to evolution has long been understood (Corning, 2014), and recent integrative studies combining genetics and behavioral ecology point toward two phenomena of particular importance to our understanding of contemporary chondrichthyan evolution. Indirect genetic effects (IGEs) occur where the genotype of one individual influences the phenotype of another (Wolf et al., 1998). These effects—which are often cryptic and difficult to detect—are important in an evolutionary context as they can modulate the response to selection (McGlothlin & Brodie III, 2009), thus acting as “pacemakers” of adaptive evolution (Bailey et al., 2018). The other phenomenon of particular importance is plasticity first evolution—the proposition that phenotypic plasticity (including behavior, which is intrinsically plastic) may precede and facilitate adaptive evolution by providing the “raw material” upon which selection can act in the absence of de novo, mutation‐based adaptations (Levis & Pfennig, 2016; Perry et al., 2018). This, like IGEs, could increase the rate at which adaptive evolution occurs, although debate exists regarding the validity of such hypotheses (Levis & Pfennig, 2016). As many chondrichthyan populations have low effective population sizes (Pazmiño et al., 2017) and long generation times (Cailliet et al., 2005), both IGEs and plasticity first adaptations could play a major role in determining the vulnerability of contemporary populations to rapid environmental change, yet until now these phenomena have been ignored in the context of chondrichthyan evolution.

This lack of integration is driven by many of the same limitations affecting integration between genetics and morphology, and as such can be overcome using broadly similar methodological approaches (Figure 1). Studies of past selection may provide valuable insights into the evolution of behavioral traits (Eusebi et al., 2018; Grams et al., 2015), and quantitative genetic studies are valuable not only for uncovering the genetic basis of these traits (Bubac et al., 2020) but for providing direct evidence of IGEs and plasticity first evolution. The major difference is that whilst morphology is easily quantified, relatively little is known about chondrichthyan behavioral ecology (Bres, 1993; Guttridge et al., 2009), and most existing studies are descriptive or qualitative in nature. This will have to be overcome before quantitative genetic methodologies can be applied, and thus I suggest that future studies of chondrichthyan behavior must focus on quantifying behavioral variation within and between populations as a matter of urgency.

## 4 LINKING MORPHOLOGY TO BEHAVIOR

Uncovering the genetic basis of behavioral and morphological traits and the factors influencing their evolution is undoubtedly of great importance, but this only provides one half of the story; it is one thing to establish the mechanisms of evolution and their phenotypic consequences, but another entirely to elucidate the functional link between them (Arnold, 1983). The associations between morphology and behavior are perhaps the best‐studied of the three integrative pathways discussed here as in a broad sense uncovering these associations is the express goal of functional morphology (Wainwright, 1994). Several experimental studies have addressed the functional significance of chondrichthyan morphology (Wilga & Lauder, 2004a), paying particular attention to the dorsal, pectoral, and caudal fins as well as dermal denticles (Feld et al., 2019; Maia & Wilga, 2013; Wilga & Lauder, 2001, 2004b). There are however several major limitations of this approach that constrain integration between morphological and behavioral studies. This experimental approach rarely captures ecologically relevant complex behaviors, and where it does, influence of the laboratory setting on expressed behavior cannot be ruled out (Moore & Biewener, 2015). The lack of studies linking morphology to complex behaviors in wild populations fundamentally constrains our understanding of evolution as without such studies the true adaptive value of morphology cannot be reliably ascertained. This in turn constrains our understanding of ecological interactions, the evolution of complex behaviors such as foraging strategies, and how future environmental change may influence them.

There has been a move toward performance‐based studies of chondrichthyan kinematics in recent years, focussing more on the ability of individuals to perform ecologically relevant behaviors (such as prey handling, or “punting” in skates) than the function of individual components of morphology (Irschick & Higham, 2016; Macesic & Kajiura, 2010; Moyer et al., 2021; Moyer & Dodd, 2023). This approach is highly beneficial given that natural selection likely acts on performance and that most (if not all) ecologically relevant behaviors require the coordinated use of multiple components of morphology (Arnold, 1983). Moreover, the prevalence of many‐to‐one mapping of form to function means that performance‐based studies are critical to our understanding of functional morphology (Wainwright, 2007; Wainwright et al., 2005). Only through a performance‐based approach can we gain a holistic understanding of the ecological selection underlying morphological evolution. Unfortunately, even in the case of performance‐based studies, population and species‐specific morphologies (Grover, 1972; Keeney & Heist, 2006; Sternes & Shimada, 2020) mean that there is little reason to suggest that existing kinematic studies should be representative of all—or even a substantial proportion of extant chondrichthyan diversity. Novel technological advancements are increasingly enabling quantitative study of chondrichthyan behavior in wild populations (Butcher et al., 2021; Renshaw et al., 2023), and thus with sufficient research effort this knowledge gap is likely to decrease, however, significant further study will be required in order to achieve this goal. I suggest that kinematic studies should wherever possible utilize a performance‐based approach, which is likely to be far more informative from an evolutionary perspective than studies focussing on individual morphological structures. In the absence of laboratory‐held populations of the majority of extant chondrichthyan taxa, a combination of modeling, observational, and performance‐based kinematic studies will be required to fully elucidate the complex relationships between morphology and behavior across chondrichthyan diversity.

## 5 CONCLUSIONS

Much progress has been made in recent years toward understanding chondrichthyan evolution and ecology. However, it is undeniable that many major knowledge gaps remain, and that our understanding of trait evolution in this clade is less robust than in many other vertebrate radiations. I argue that the key driver of this uncertainty is a lack of integration between genetic, morphological, and behavioral studies. Failure to integrate these key areas of organismal biology fundamentally constrains our understanding of phenotypic evolution, both past and present. Most significantly, this impedes the study of the genetic architectures underlying phenotypes and how selection acts upon them. Despite this, the necessary tools to overcome this major limitation already exist and have been applied to other taxa (Figure 1). Future studies should focus on increasing the taxonomic breadth of existing studies of chondrichthyan genetics, morphology, and behavior, as well as the implementation of more quantitative genetic and evo‐devo approaches. Only through this will we truly be able to understand trait evolution in Chondrichthyes to a comparable extent to other major vertebrate radiations, and its implications for past evolution and vulnerability in the face of climate change.

## AUTHOR CONTRIBUTIONS

**Joel H. Gayford:** Conceptualization (lead); writing – original draft (lead); writing – review and editing (lead).

## FUNDING INFORMATION

The author declares no funding sources for this study.

## CONFLICT OF INTEREST STATEMENT

The author declares no competing interests.

## ETHICAL APPROVAL

No experimental work was carried out at any point during this study, either involving animals or otherwise.

## Data Availability

Data sharing not applicable to this article as no datasets were generated or analysed during the current study.
